# Coralliform cataract caused by a novel connexin46 (*GJA3*) mutation in a Chinese family

**Published:** 2012-01-25

**Authors:** Xiaohui Zhang, Lina Wang, Jun Wang, Bing Dong, Yang Li

**Affiliations:** 1 Beijing Institute of Ophthalmology, Beijing Tongren Eye Center, Beijing Tongren Hospital, Capital Medical University, Beijing Ophthalmology & Visual Sciences Key Laboratory, Beijing, China; 2 Department of Ophthalmology, Beijing Tiantan Hospital, Capital Medical University, Beijing, China

## Abstract

**Purpose:**

To identify a novel disease-causing mutation of the *GJA3* (gap junction alpha-3 protein) gene in a Chinese family with autosomal dominant congenital cataract (ADCC).

**Methods:**

One family was examined clinically. After informed consent was obtained, genomic DNA was extracted from the venous blood of all participants. Genetic linkage analysis was performed on the known genetic loci for ADCC with a panel of polymorphic markers, and then mutations were screened by direct sequencing. Whenever substitutions were identified in a patient, high-resolution melt curve analysis (HRM) was performed on all available family members and 100 normal controls. Bioinformatics analysis was undergone by the Garnier-Osguthorpe-Robson (GOR) and the PolyPhen (polymorphism phenotyping) programs to predict the effect of variants detected on secondary structure and protein function of the GJA3 protein.

**Results:**

Clinical examination and pedigree analysis revealed one four-generation family with congenital nuclear coralliform cataracts. Significant two-point LOD (linkage odd disequilibrium) score was generated at marker D13S292 (Z_max_=2.51, θ=0), and further linkage and haplotype studies confined the disease locus to 13q11–13. Mutations screening of *GJA3* in this family revealed an A→T transversion at position 563 (p.N188I) of the cDNA sequence. This novel missense mutation co-segregated with the affected members of the pedigree, but is not present in the unaffected relatives or 100 normal individuals. Secondary structure prediction suggested that the mutant GJA8 188I would replace three turns “T” with three β sheet “E” at amino acid 189–191 and a β sheet “E” with a turn “T” at position 194.

**Conclusions:**

Novel missense mutation in the second extracellular loop (E2) was detected, causing coral-like opacities involving embryonic and fetal nucleus surrounded by blue punctate opacities in the cortical zone of the lens. The results further suggested that the extracellular loop was the mutation hotspot of GJA3.

## Introduction

Cataract, defined as any opacity of the crystalline lens, can be divided into early onset (congenital or infantile) and age-related cataract. Congenital cataract with a prevalence round of 1–6 cases per 10,000 live births, is an important cause of blindness in children [1]. Approximately one-third of congenital cataracts are hereditary and most of them often occur in a nonsyndromic autosomal dominant fashion [2]. Congenital cataract exhibits high clinical and genetic heterogeneity. To date, at least 30 independent loci for autosomal dominant congenital cataract (ADCC) have been mapped on human chromosomes and 20 genes have been implicated in human cataractogenesis [1-3]. Of the disease-causing mutations found in the cataract families, about half are identified in crystallins, a quarter in connexins, and the remainder divided among the genes for heat shock transcription factor-4 (*HSF4*), aquaporin-0 (*AQP0*, MIP), and beaded filament structural protein-2 (*BFSP2*) [3].

In humans, there are 21 different connexin genes that exhibit complex and overlapping patterns of expression [4]. Each connexin gene codes for a transmembrane connexin protein with the same protein topology. Six connexin proteins oligomerize to form hemichannels (connexons), which then are transported to the plasma membrane. Hemichannels from two adjacent cells align in the extracellular space to complete the formation of a cell-to-cell channel (gap junction). There are three connexin genes (gap junction alpha-1 protein [*GJA1*], gap junction alpha-3 protein [*GJA3*], and gap junction alpha-8 protein [*GJA8*]) expressed in the human lens. The epithelial cells on the anterior surface of the human lens express *GJA1* and *GJA8*, while the specialized lens fibers, which constitute the majority of the organ, express *GJA3* and *GJA8* [4,5]. *GJA3*, located on chromosome 13q11, encodes a protein containing 435 amino acids [6]. Like other connexin proteins, connexin 46 (Cx46) consists of four transmembrane domains (TM1–TM4), two extracellular loops (E1 and E2), a cytoplasmic loop (CL) between TM2 and TM3, and cytoplasmic N-terminal (NT) and C-terminal (CT) domains. So far, 17 mutations have been identified in the *GJA3* gene and more than half of them are located in the two extracellular loops [6-20].

This study investigated a Chinese family with coralliform cataracts. After linkage analysis, we mapped the disease-causing gene to the locus 13q11 where connexin 46 (*GJA3*) is located and detected a novel missense mutation.

## Methods

### Clinical data and sample collection

This study adhered to the tenets of the Declaration of Helsinki for research involving human subjects. The Beijing Tongren Hospital Joint Committee on Clinical Investigation approved the study. One Chinese family with congenital cataracts was referred to Beijing Tongren Hospital. After informed consent was obtained, each participant underwent careful ophthalmologic examinations, including best-corrected visual acuity testing using E decimal charts, slit-lamp biomicroscopy, and fundus examination with dilated pupils. Peripheral blood was obtained by venipuncture, and genomic DNA was extracted according to standard phenol protocols.

### Genotyping and linkage analysis

Genotyping was performed with 22 microsatellite markers from autosomes for the known ADCC loci in this family (Appendix 1). Then, genotyping and linkage analysis was performed with another two microsatellite markers, D13S1236 and D13S292, around the *GJA3* gene. The fine mapping primer sequences were obtained from the Human Genome Database (GDB). LOD (linkage odd disequilibrium) scores were calculated for the markers by two-point linkage analysis using linkage package 5.2. We modeled the disease as an autosomal dominant trait with 100% penetrance. Pedigree and haplotype maps were constructed using Cyrillic V. 2.0 software.

### Mutation screening of *GJA3*

We used three pairs of primers to amplify the sole coding region of *GJA3* by polymerase chain reaction (PCR) from genomic DNA (Table 1). For direct sequencing, amplicons were purified (Shenneng Bocai PCR purification kit; Shenneng, Shanghai, China). An automatic fluorescence DNA sequencer (ABI, Prism 373A; Perkin Elmer, Foster City, CA), used according to the manufacturer’s instructions, sequenced the purified PCR products in both forward and reverse directions. Nucleotide sequences were compared with the published cDNA sequence of *GJA3* (GenBank NM_021954.3). For *GJA3*, cDNA numbering +1 corresponds to A in the ATG translation initiation codon of *GJA3*.

**Table 1:** Primer information for *GJA3* gene sequencing.

**Primer**	**Forward sequence (5′-3′)**	**Reverse sequence (5′-3′)**	**Products (bp)**	**Tm (°C)**
Fragment 1	CGGTGTTCATGAGCATTTTC	GTCTTGAAGATGATGTTGAA	470	60
Fragment 2*	GGCGCTGCTGCGGACCTACG	CGTCCGGGCCGAGGCGGCTG	296	58
Fragment 3	AAGCTCAAGCAGGGCGTGAC	TATCTGCTGGTGGGAAGTGC	708	60

* Primer used in high resolution melt curve analysis (HEM) in this study.

### High-resolution melt curve analysis (HRM)

To confirm the variation found in the sequencing, high-resolution melt curve analysis (HRM) was performed in the available family members and in 100 normal controls. The 10 μl reaction mixture consisted of 5 μl SsoFast EvaGreen Supermix (Bio-Rad Laboratories, Inc., Hercules, CA), 1 μl genomic DNA (10–150 ng/μl), 0.5 μl forward primer (10 pmol/μl), 0.5 μl reverse primer (10 pmol/μl), and 3 μl double distilled water. Polymerase chain reaction (PCR) cycling and HRM analysis were performed on the Rotor-Gene 6000^TM^ (Corbett Research, Mortlake, New South Wales, Australia) [21].

### Bioinformatics analysis

Garnier-Osguthorpe-Robson (GOR) software was used to predict the effect of the mutation on the secondary structure of GJA3 [22]. This method infers the secondary structure of a sequence by calculating the probability for each of the four structure classes (helix, sheet, turn, and loop) based on the central residue and its neighbors from the calculated matrices [22]. The PolyPhen (polymorphism phenotyping) program was used to predict the potential functional impact of an amino acid change. A PolyPhen score above 2.0 is predicted as probably damaging to protein function, a PolyPhen score between 1.5 and 2.0 is considered possibly damaging, while PolyPhen scores below 1.5 are considered likely benign [23].

## Results

### Clinical findings

**Figure 1:**
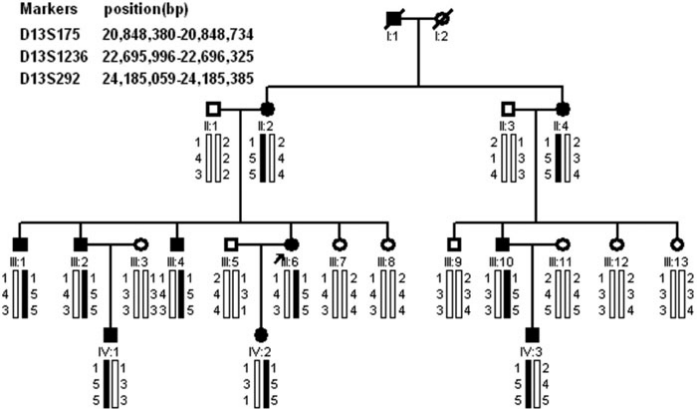
Family structure and haplotype analysis of a Chinese family with congenital cataract. Pedigree and haplotype analysis of the family with congenital cataract showed segregation with three microsatellite markers on chromosome 13 listed in descending order from the centromeric end. Squares indicate males; circles indicate females; slashed symbols indicted deceased; solid symbols indicate affected; open symbols indicate unaffected.

**Figure 2:**
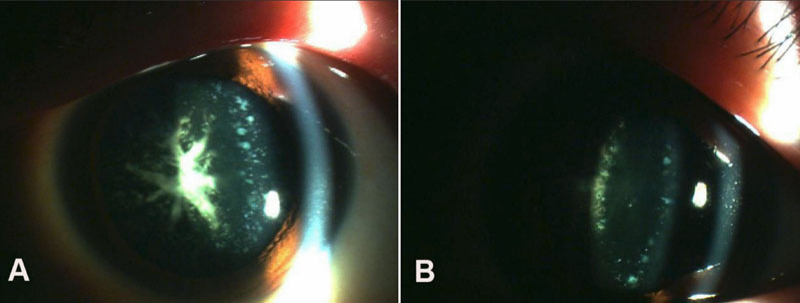
Slit-lamp photograph of the right eye of proband (III:6) in this four-generation Chinese family. **A**: Slit-lamp photograph showed dense coral-like opacities located in the central zone of the lens. **B**: Fine blue punctate opacities in the cortical zone were also observed in the same eye.

We have identified a four-generation family diagnosed with congenital cataract (Figure 1). After reviewing clinical examinations and hospital records, 11 individuals were found to have bilateral congenital cataracts and nine of them had had a cataract extraction before the examination. The remaining two patients presented with almost the same appearance of cataracts, consisting of dense coralliform opacities in the central or nuclear region of the lens and fine blue dust-like opacities in the cortical zone (Figure 2).

### Genotyping results

First, this family was genotyped with 22 polymorphic markers around the known ADCC loci. The mapping results excluded the other known ADCC loci with the exception of the *GJA3*.Two-point LOD score for D13S175 was 1.60 (θ=0) with full penetrance. In the further linkage analysis, the LOD scores for the markers D13S292 and D13S1236 were 2.51 (θ=0.0) and 2.39, respectively. Linkage and haplotype analysis for these three markers suggested that the *GJA3* gene might be the disease-causing gene of this family (Figure 1).

### Mutation analysis

**Figure 3:**
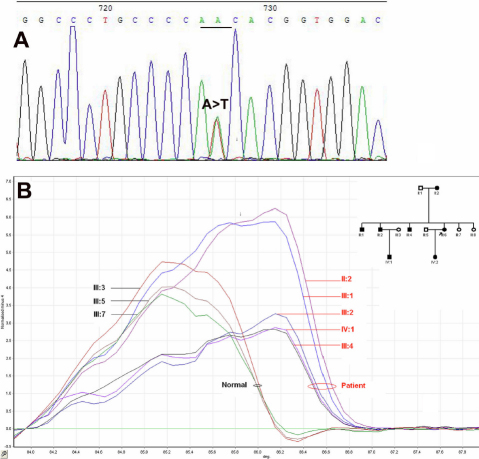
DNA sequence chromatograms and cosegregation analysis of the p.N188I mutation with disease phenotype. **A**: Heterozygote sequence (sense strand) shows an A/T transition in codon 188 that changed asparagine (AAC) to isoleucine (ATC). **B**: A difference plot of the eight members in this family by high-resolution melt curve analysis (HRM) for the mutation p.N188I. The median green, straight line presents the normal control line. The real-time PCR products of the family members are compared to the median normal control to produce the plot. The curve revealed that the mutant pattern (red ring pointed) co-segregated with the affected individuals, but not with the unaffected individuals (black ring pointed).

By direct sequencing of only one coding exon of *GJA3*, we found a novel base change (A→T) at position 563 of the *GJA3* cDNA, replacing asparagine with isoleucine at amino acid 188 residue (Figure 3A). Using HRM analysis, this novel mutation (p.N188I) co-segregated with all affected members in this Chinese family, but was not detected in 100 unrelated normal controls (Figure 3B).

### Bioinformatics analysis

**Figure 4:**
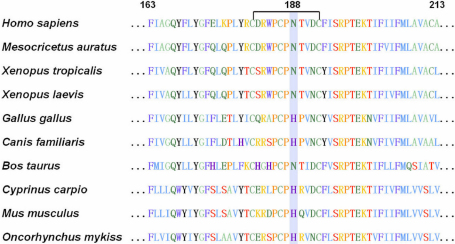
Sequence alignment portion of the second extracellular loop domain spanning the novel missense mutation p.N188I of human GJA3 with other species.

Using the GOR method, the results for secondary structure prediction suggested that the mutant GJA8 188I replaced three turns “T” with three β sheet “E” at amino acid 189–191 and a β sheet “E” with a turn “T” at position 194 (Figure 4). By PolyPhen program analysis, the PolyPhen score was 2.243 for the p.N188I, which meant that this mutation is probably damaging.

## Discussion

In this study, we mapped a four-generation Chinese congenital pedigree to the *GJA3* locus and identified a novel missense mutation, p.N188I. This substitution co-segregated with the phenotype of this pedigree, but was not detected in 100 normal controls.

**Figure 5:**
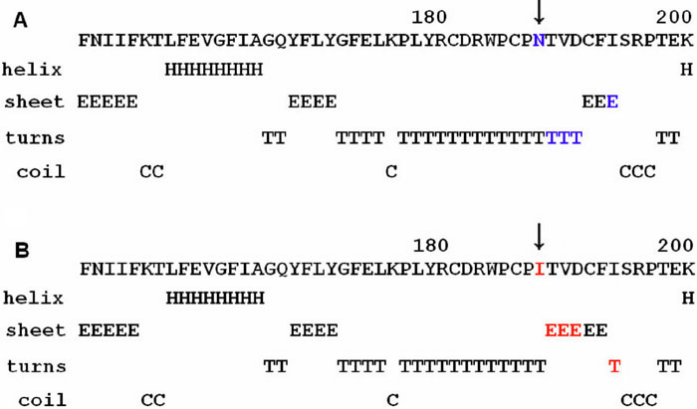
The effect of p.N188I on secondary structure of GJA3 using the GOR method. **A**: The secondary structure of wild type GJA3 round the site N188 (in blue). **B**: The secondary structure of mutant p.N188I (in red) of GJA3 of the corresponding region.

The Asn188 residue is located in a phylogenetically conserved motif of 12 amino acids, which contains three cysteine residues (181-CDRWPCPNTVDC-192), within the second extracellular loop domain (E2; Figure 4). The result of GOR analysis suggested that p.N188I led to significant secondary structure changes between residue 189 and 194, which may interfere with the correct folding of the second extracellular loop (Figure 5). In our previous study, another mutation, p.N188T, was also detected in Asn188 residue in a large Chinese family [10]. Both the E1 and E2 domains are believed to function in regulating cell-cell recognition and docking of connexin hemi-channels (connexin hexamers) via cysteine-cysteine disulfide bridges in the intercellular space [4,5]. The mutation p.N63S, reported by Mackay et al. [6], is located in another phylogenetically conserved motif (54-CNTQQPECENVC-65) of the first extracellular loop (E1) domain. The functional expression studies have shown that the wild type human Cx46 formed functional gap-junction channels in paired *Xenopus* oocytes and hemi-gap-junction channels in single oocytes; however, the mutant p.N63S formed neither of them [24]. In our review of the literature, nine mutations of Cx46 lie in the two extracellular loops. Six of them are located in the two conserved motifs of 12 amino acids containing three cysteine residues; however, no mutation has been detected in the exact one of the six cysteine residues (Table 2).

**Table 2:** Summary of reported mutations in GJA3 with different congenital cataract phenotypes.

**Mutation**	**Location**	**Cataract type**	**Phenotype description**	**Family origin**	**Reference**
p.D3Y	NH_2_-terminus	Zonular pulverulent	Progressive zonular pulverulent cataract	Hispanic	[14]
p.L11S	NH_2_-terminus	Ant-egg	lamellar cataract with dense pearl-like or ant-egg-like structures imbedded in the lens	Danish	[15]
p.V28M	First transmembrane domain (TM1)	Variable	Total or anterior capsular with posterior cortical opacitiey in different affect persons	Indian	[12]
p.F32L	First transmembrane domain (TM1)	Nuclear pulverulent	Punctate opacities in the central zone of the lens limited to the embryonal nucleus	Chinese	[8]
p.R33L	First transmembrane domain (TM1)	Embryonal nuclear granular	a band of numerous granular opacities involving the embryonal nucleus	Indian	[17]
p.V44M	First extracellular loop (EL1)	Nuclear	central nuclear opacity involving embryonic and fetal nucleus with punctate cortical opacities	Chinese	[18]
p.W45S	First extracellular loop (EL1)	Nuclear	Progressive nuclear cataract	Chinese	[13]
p.D47N	First extracellular loop (EL1)	Nuclear	central nuclear opacity affecting the embryonic and fetal nucleus of the lens	Chinese	[19]
p.P59L	First extracellular loop (EL1)	Nuclear punctate	Coarse opacities located in the central region of the lens	American	[9]
p.N63S	First extracellular loop (EL1)	Zonular pulverulent	Fine dust-like opacities in the central region of the lens	Caucasian	[6]
p.R76G	Boundary of EL1 and TM2	Total	Total opacitiey of the lens	Indian	[12]
p.R76H	Boundary of EL1 and TM2	Zonular pulverulent	Faint lamellar nuclear opacity surrounding pulverulent nuclear opacityis with gold dots or fine haze or needle-like peripheral riders	Australian	[11]
p.T87M	Second transmembrane domain (TM2)	Pearl box	Fine white spots made up the opacity of the fetal nucleus . The embryonalnucleus was composed of coarse white spots of various sizes.	Indian	[16]
p.P187L	Second extracellular loop (EL2)	Zonular pulverulent	“Dust-like opacity affectingthe embryonal,fetal and infantile nucleuws of lesns surrounded by snowflake-like opacities in the anterior and posterior cortical region of the lens”	Caucasian	[7]
p.P187S	Second extracellular loop (EL2)	Nuclear pulverulent	central nuclear opacity with punctate opacities	Chinese	[20]
p.N188T	Second extracellular loop (EL2)	Nuclear pulverulent	Progressive zonular pulverulent cataract affecting the embryonic and fetal nucleus of the lens	Chinese	[10]
p.N188I	Second extracellular loop (EL2)	Zonular pulverulent	coral-like nuclear opacity involving embryonic and fetal nucleus surrounded by blue punctate cortical opacities	Chinese	Present study
p.S380fs	COOH-terminus	Zonular pulverulent	Coarse and granular opacities located in the central region of the lens.Fine dust-like opacities predominated in the peripherial zone of the lens.	Caucasian	[6]

So far, 17 different mutations in *GJA3* have been reported [6-20]. All mutations, with one exception, p.S380fs located in the COOH-terminus of Connexin 46, are missense mutations (Table 2). Clinically, the Cx46 related cataracts share several genotype-phenotype similarities with inter- and intra-familial differences with respect to the morphological opacities of the lens and location of opacities (Table 2). Most of the cataract phenotypes are of nuclear or zonular pulverulent types. However, in the present study, dense white coral-form opacity was observed in the embryonic and fetal nucleus, which were surrounded by diffuse blue punctate opacity in the cortical region of the lens. Coralliform cataract is a special form of congenital cataract, which has been observed in several ADCC families carrying *CRYGD* mutations [25-30]. Mutation p.P23T of CRYGD is the most common cause of coralliform cataract [25-28], the mutations p.R14C and p.G61C can also cause coralliform cataract [29,30]. The resemblance in the morphologies of cataract phenotypes associated with different genes mutations (or different mutations of the same gene) may due to the interaction of background environmental and/or the action of modifier genes.

In conclusion, we identified a novel mutation of *GJA3* in a Chinese family with congenital cataract. Our findings further suggest that the extracellular loop is the mutation hotspot of the GJA3.

## Appendix 1. Markers used in the known ADCC genotyping.

To access the data, click or select the words “Appendix 1.” This will initiate the download of a compressed (pdf) archive that contains the file.

## References

[1] Francis PJ, Berry V, Bhattacharya SS, Moore AT (2000). The genetics of childhood cataract.. J Med Genet.

[2] Reddy MA, Francis PJ, Berry V, Bhasttacharya SS, Moore AT (2004). Molecular genetic basis of inherited cataract and associated phenotypes.. Surv Ophthalmol.

[3] Hejtmancik JF (2008). Congenital Cataracts and their Molecular Genetics.. Semin Cell Dev Biol.

[4] Pfenniger A, Wohlwend A, Kwak BR (2011). Mutations in connexin genes and disease.. Eur J Clin Invest.

[5] Mathias RT, White TW, Gong X (2010). Lens gap junctions in growth, and homeostasis differentiation.. Physiol Rev.

[6] Mackay D, Ionides A, Kibar Z, Rouleau G, Berry V, Moore A, Shiels A, Bhattacharya S (1999). Connexin 46 mutations in autosomal dominant congenital cataract.. Am J Hum Genet.

[7] Rees MI, Watts P, Fenton I, Clarke A, Snell RG, Owen MJ, Gray J (2000). Further evidence of autosomal dominant congenital zonular cataracts linked to 13q11 (CZP3) and a novel mutation and a novel mutation in connexin 46 (GJA3).. Hum Genet.

[8] Jiang H, Jin Y, Bu L, Zhang W, Liu J, Cui B, Kong X, Hu L (2003). A novel mutation in GJA3 (connexin 46) for autosomal dominant congenital nuclear pulverulent cataract.. Mol Vis.

[9] Bennett TM, Mackay DS, Knopf HLS (2004). Shiels A. A novel missense mutation in the gene for gap junction protein (GJA3) associated with autosomal dominant “nuclear punctate”cataracts linked to chromosome 13q.. Mol Vis.

[10] Li Y, Wang J, Dong B, Man H (2004). A connexin 46 (GJA3) mutation in autosomal dominant congenital nuclear pulverulent cataract.. Mol Vis.

[11] Burdon KP, Wirth MG, Mackey DA, Russell-Eggitt IM, Craig JE, Elder JE, Dickinson JL, Sale MM (2004). A novel mutation in the connexin 46 gene causes autosomal dominant congenital cataract with incomplete penetrance.. J Med Genet.

[12] Devi RR, Reena C, Vijayalakshmi P (2005). Novel mutations in GJA3 associated with autosomal dominant congenital cataract in the Indian population.. Mol Vis.

[13] Ma ZW, Ma Z, Zheng JQ, Li J, Li XR, Tang X, Yuan XY, Zhang XM, Sun HM (2005). Two novel mutations of connexin genes in Chinese families with autosomal dominant congenital nuclear cataract.. Br J Ophthalmol.

[14] Addison PK, Berry V, Holden KR, Espinal D, Rivera B, Su H, Srivastava AK, Bhattacharya SS (2006). A novel mutation in the connexin 46 gene (GJA3) causes autosomal dominant zonular pulverulent cataract in a Hispanic family.. Mol Vis.

[15] Hansen L, Yao W, Eiberg H, Funding M, Riise R, Kjaer KW (2006). Hejtmancik JF, Rosenberg T. The congenital “ant-egg” cataract phenotype is caused by a missense mutation in connexin 46.. Mol Vis.

[16] Guleria K, Vanita V, Singh D, Singh JR (2007). A novel “pearl box” cataract associated with a mutation in the connexin 46 (GJA3) gene.. Mol Vis.

[17] Guleria K, Sperling K, Singh D, Varon R, Singh JR, Vanita V (2007). A novel mutation in the connexin 46 (GJA3) gene associated with autosomal dominant congenital cataract in an Indian family.. Mol Vis.

[18] Zhou Z, Hu S, Wang B, Zhou N, Zhou S, Ma X, Qi Y (2010). Mutation analysis of congenital cataract in a Chinese family identified a novel missense mutation in the connexin 46 gene (GJA3).. Mol Vis.

[19] Yang G, Xing B, Liu G, Lu X, Jia X, Lu X, Wang X, Yu H, Fu Y, Zhao J (2011). A novel mutation in the GJA3 (connexin 46) gene is associated with autosomal dominant congenital nuclear cataract in a Chinese family.. Mol Vis.

[20] Ding X, Wang B, Luo Y, Hu S, Zhou G, Zhou Z, Wang J, Ma X, Qi Y (2011). A novel mutation in the connexin 46 (GJA3) gene associated with congenital cataract in a Chinese pedigree.. Mol Vis.

[21] Maltese P, Canestrari E, Palmaa L, Ruzzo A, Corini F, Menotta M, Andreonic F, Latiano A, Annese V, Magnani M (2009). High-resolution melting (HRM) analysis for the detection of ER22/23EK, BclI, and N363S polymorphisms of the glucocorticoid receptor gene.. J Steroid Biochem Mol Biol.

[22] Garnier J, Gibrat JF, Robson B (1996). GOR method for predicting protein secondary structure from amino acid sequence.. Methods Enzymol.

[23] Ramensky V, Bork P, Sunyaev S (2002). Human non-synonymous SNPs: server and survey.. Nucleic Acids Res.

[24] Pal JD, Liu X, Mackay D, Shiels A, Berthoud VM, Beyer EC, Ebihara L (2000). Connexin 46 mutations linked to congenital cataract show loss of gap junction channel function.. Am J Physiol Cell Physiol.

[25] Mackay DS, Andley UP, Shiels A (2004). A missense mutation in the gammaD crystallin gene (CRYGD) associated with autosomal dominant “coral-like” cataract linked to chromosome 2q.. Mol Vis.

[26] Xu WZ, Zheng S, Xu SJ, Huang W, Yao K, Zhang SZ (2004). Autosomal dominant coralliform cataract related to a missense mutation of the gammaD-crystallin gene.. Chin Med J (Engl).

[27] Khan AO, Aldahmesh MA, Ghadhfan FE (2009). Founder heterozygous P23T CRYGD mutation associated with cerulean (and coralliform) cataract in 2 Saudi families.. Mol Vis.

[28] Yang G, Xiong C, Li S, Wang Y, Zhao J (2011). A recurrent mutation in CRYGD is associated with autosomal dominant congenital coralliform cataract in two unrelated Chinese families.. Mol Vis.

[29] Gu F, Li R, Ma XX, Shi LS, Huang SZ, Ma X (2006). A missense mutation in the? D-crystallin gene GRYGD associated with autosomal dominant congenital cataract in a Chinese family.. Mol Vis.

[30] Li F, Wang S, Cao C, Liu S, Zhao B, Zhang M, Huang S, Zhu S, Ma X (2008). Mutation G61C in the CRYGD gene causing autosomal dominant congenital coralliform cataracts.. Mol Vis.

